# I’m not just fat, I’m old: has the study of body image overlooked “old talk”?

**DOI:** 10.1186/2050-2974-1-6

**Published:** 2013-02-21

**Authors:** Carolyn Black Becker, Phillippa C Diedrichs, Glen Jankowski, Chelsey Werchan

**Affiliations:** 1 Department of Psychology, One Trinity Place, Trinity University, 78212, San Antonio, TX, USA; 2 Center for Appearance Research, University of the West of England, Coldharbour Lane, BS16 1QY, Bristol, UK

**Keywords:** Old talk, Fat talk, Body image, Body dissatisfaction, Eating disorders, Self objectification, Ageing, Ageing anxiety

## Abstract

**Background:**

Research indicates that body dissatisfaction is correlated with and often predictive of both physical and mental health problems. “Fat talk,” a well-studied form of body image talk in adolescents and university-aged women, has been implicated as contributing to body dissatisfaction and mediating the relationship between body dissatisfaction and other mental health problems. Limited research, however, has investigated fat talk across the female lifespan. Further, consistent with most body image research, fat talk research solely focuses on the thin dimension of idealized female attractiveness, even though other dimensions may contribute to body dissatisfaction in women.

**Method:**

The current study investigated whether or not “old talk,” a hereto un-described form of body image talk, appears to be a parallel, but distinct, form of body image talk that taps into the young dimension of the thin-young-ideal standard of female beauty. An international, internet sample of women (aged 18-87, *N* = 914) completed questionnaires aimed at assessing fat talk, old talk, body image disturbance, and eating disorder pathology.

**Results:**

Results indicated that both fat talk and old talk were reported by women across the lifespan, although they evidenced different trajectories of frequency. Like fat talk, old talk was significantly correlated with body image disturbance and eating disorder pathology, albeit at a lower rate than fat talk in the total sample. Old talk was more highly correlated with ageing appearance anxiety than fat talk, and the correlation between old talk and body image disturbance and ED pathology increased with women’s ages.

**Conclusion:**

Results suggest that old talk is a form of body image talk that is related to but distinct from fat talk. Old talk appears to be similarly problematic to fat talk for women whose age increases their deviation from the thin-young-ideal. Further research into the phenomenon of old talk is warranted as is increased attention to fat talk across the full lifespan of women.

## Background

Substantial research indicates that body dissatisfaction broadly defined is highly correlated with, and often longitudinally predictive of, both physical and mental health problems [1,2]. For example, among adolescent girls, researchers have found that body dissatisfaction (e.g., as measured by standardized questionnaires such as the Body Shape Satisfaction Scale [3]) prospectively predicts increases in binge eating, emotional eating, stress, low self-esteem, depression, use of unhealthy weight control behaviors, decreased physical activity, and, among overweight girls, increased weight gain between 1-5 years later [4-6]. Although most research on body dissatisfaction has been conducted with females, existing research, which is largely cross-sectional, suggests that body dissatisfaction in males is associated with eating disorders (ED), steroid use, poor psychological adjustment, and exercise dependence [7].

### Ageing and body image

In addition to disproportionally targeting females, past body image research also has largely focused on a narrow age range, namely adolescents and university-aged women [8]. This is despite the fact that there is little evidence to suggest that graduation from university is associated with a graduation from body image concerns. The limited research on body image in older women suggests that body dissatisfaction is relatively comparable in younger and older women [8-11]. Cross-sectional research also supports a relationship between body dissatisfaction in adult women and depression, decreased quality of life, fewer pleasant feelings, increased negative feelings, increased ED pathology, and decreased self-care [12,13]. Finally, body dissatisfaction in adult women smokers prospectively predicts greater difficulty quitting smoking [14].

There are reasons to think that body dissatisfaction might actually increase as women age [8]. For example, normal ageing typically moves women’s body weight/shape further away from the thin-ideal standard of female beauty in Western culture. Moreover, as women age, they encounter a variety of developmental stages that may accelerate this movement away from the thin-ideal. Some examples include pregnancy, decreased time for exercise and self-care secondary to demands of work, home, and/or childcare, menopause, and possibly the use of medications that may increase weight. Demands on young adult and midlife women to conform to the thin-ideal also have increased with the media’s focus on pregnant celebrity women, who are lauded for losing weight post-pregnancy quickly and returning to their “pre-baby” bodies (e.g., http://thestir.cafemom.com/pregnancy/144512/7_celebrity_moms_whose_postbaby). Adding to this pressure is the fact that the thin-ideal is also a young-ideal. As evidence of this, there has been a proliferation of interest in, and advertising of, anti-ageing cosmetic products, pharmaceuticals and surgical procedures, all of which are marketed to women with the underlying message that a youthful, wrinkle-free appearance is ideal [15]. Similarly, models depicted in the media are predominantly thin and young, with a recent content analysis of advertisements in popular North American women’s magazines concluding that 80.72% of the models were aged 18-30 years [16]. Thus, as women age, they increasingly move away not just from being thin but also from fulfilling the young element of the thin-young-ideal. Accordingly, ageing creates new opportunities for discrepancies between women’s bodies and cultural beauty ideals.

Indeed, cross-sectional research supports a relationship between ageing anxiety and both body dissatisfaction and EDs [17]. As such, the relative stability of body dissatisfaction across age groups is, in some ways, surprising. Some evidence, however, suggests that older women may more frequently employ cognitive strategies to partially offset their increasing distance from our societies’ conceptualization of the ideal woman [8,11,12]. It also may be that the nature of women’s body image concerns change and that they have not been adequately assessed by approaches that were developed with, and for, younger women. Lastly, there may be cohort effects. More specifically, researchers may increasingly find higher levels of body dissatisfaction in older women over time as these women have grown up in a more toxic body image climate than those of previous generations.

We should note that although most research suggests that cross-sectional samples across the female lifespan report similar levels of body dissatisfaction on standardized measures, a few studies indicate that body dissatisfaction is slightly lower in some (but not all) domains for women in older adulthood (i.e., over age 60) [8,13,18-20]. For instance, in a large cross-sectional sample of women over 50, being older was associated with decreased concern in certain facets of body image (e.g., that weight or shape negatively affected life, lesser importance of weight or body shape on self-perception), but not others (e.g. being moderately to extremely upset with gaining five pounds [18]). In summary, women across the lifespan appear to report similarly elevated levels of dissatisfaction with their bodies, although women in older adulthood (60 years and older) may report less dissatisfaction in some domains such as a perception that weight and shape negatively affected life, in comparison to younger women [18].

### Fat talk and old talk

Numerous sociocultural, intrapersonal and interpersonal factors contribute to girls and women’s body dissatisfaction. One factor that has received increasing attention is “fat talk” (e.g., “I’m so fat,” “Wow, you look great, have you lost weight?”). The term fat talk was coined by Nichter and Vuckovic [21,22] to describe the body image talk in which pre- and adolescent girls engage when talking about the size and shape of their bodies. Nichter and Vukovic reported that this population used fat talk for impression management (i.e., to increase social likeability and decrease perceptions of arrogance). Fat talk is hypothesized to increase a sense of inter-connectedness between girls and women; yet at the same time, fat talk reinforces the thin-ideal and decreases the opportunity for girls to interact in more meaningful ways.

Empirical research has since documented that fat talk also is common in college-aged women [23,24] and pernicious. In cross-sectional samples, fat talk is associated with body dissatisfaction, drive for thinness, internalization of the thin-ideal, and ED pathology [23-25]. Clarke, Murnen, and Smolak [26] found that fat talk predicted unique variance in ED symptoms and body shame. Further, Stice, Maxfield and Wells [27] found that a mere 3-5 minutes of exposure to fat talk significantly increased body dissatisfaction among undergraduate women, as compared to exposure to non-appearance-related conversations. Finally, Arroyo and Harwood [28] found evidence that fat talk explains a significant amount of variance in body dissatisfaction, perceived pressure to be thin and depression. This research also found that FT longitudinally mediated the relationship between negative body image and broader mental health concerns.

To our knowledge, only one study examined fat talk in an older sample (i.e., *M* age = 45). Martz, Petroff, Curtin, and Bazzini [29] found that women reported that, in their own lives, situations involving fat talk were significantly more likely to occur as compared to situations involving self-accepting or positive body talk. Thirty-one percent of women reported high exposure to fat talk and the mean rating of fat talk exposure was 2.74 with a score of 3 being equated to “usually”. Although Martz et al. [29] comment that these frequencies seem relatively low, the findings by Stice et al. [27] suggest that fairly low levels of exposure to fat talk can have a significant, negative effect. Indeed, Stice et al. [27] stated that it was “noteworthy that such a brief and subtle manipulation” yielded an effect of medium size (p. 113).

In 2008, the Tri Delta sorority launched Fat Talk Free® Week, aimed at raising awareness of fat talk and encouraging people to stop fat talk. Since 2008, this advocacy campaign has become both annual and international, and various locations have been declared Fat Talk Free® Zones. In 2011, the owner of a pilates studio that was a dedicated Fat Talk Free® Zone contacted the first author and asked what she was supposed to do about problematic “old talk” (e.g., “I look so old”, “Look at these wrinkles”, “Do you want to come to a Botox party?”), in which women of a variety of ages engaged (e.g., women in their 30’s, 40’s and older). In response to this, we began listening to conversations around us and in the media. We also searched the literature for any references to old or ageing talk and its relationship to body image. Although we observed the phenomenon described by the studio owner, we were unable to find any social science literature specifically pertaining to this form of body talk behavior. We hypothesized that old talk is a distinct but, until now, unstudied form of body image talk, and therefore concluded that it was worthy of at least a preliminary investigation.

### Current study

The primary aim of this mixed-methods study was to a) assess the self-reported frequency of old talk, b) gain a preliminary understanding of the content of old talk, and c) investigate whether it, like fat talk, is associated with negative body image, self-objectification, and ED pathology. Given that body image is multi-dimensional, we chose to assess several different facets of body image that have been studied in connection to fat talk including body satisfaction, thin-ideal internalization, drive for thinness as well as the related construct of self-objectification. We also assessed anxiety related to aging appearance. Because only one paper has examined fat talk in a broad age range and we needed to study old talk across the female adult lifespan, we also sought to investigate fat talk in the same sample and to compare the two forms of body image talk. Finally, since the overwhelming majority of fat talk research has been conducted within the United States (USA), we expanded the geographical boundaries of our study to the United Kingdom (UK) and Australia. We outline our specific hypotheses for this study below.

With regards to fat talk we hypothesized that: 1) fat talk would be a common occurrence (i.e., that we would find fat talk across all ages, and rates of fat talk would remain largely unchanged across age groups), 2) consistent with Salk and Englen-Maddox’s [24] failure to find a correlation between fat talk and weight, we would find fat talk across women of all body weights, 3) we would find a third person effect for frequency of fat talk in friends, family and the media based on Salk and Englen-Maddox’s [24] finding the participants reported more fat talk in others versus themselves.

With regards to old talk, we hypothesized that: 1) participants would report engaging in old talk, though at a lower rate than fat talk since younger women naturally conform to the young part of the thin-young-ideal, 2) that old talk would predominantly occur among women aged 30 and older and would increase significantly with age, 3) that we would find third person effects for friends, family and the media.

Regarding correlations with body image, self-objectification, and ED related variables, we hypothesized that: 1) since both fat talk and old talk represent some degree of buy-in to the thin-young-ideal, both would be significantly correlated with body image, self-objectification, and ED pathology measures, 2) fat talk and old talk would correlate with each other, but not perfectly, suggesting that fat talk and old talk are related but not identical constructs, 3) we would consistently find higher correlations with fat talk, as compared to old talk, for all body image, self-objectification, and ED measures except appearance ageing anxiety, which would correlate more closely with old talk. The rationale for hypothesis one is that both are likely associated with internalization of the thin-young ideal. We had no hypotheses about the qualitative data. Our primary goal was to determine if we found similar fat talk content to that reported by Salk and Englen-Maddox [24] in their mixed-methods fat talk study with college women and to see if old talk content was similar to what we found for fat talk or not.

## Methods

### Participants

A snowballing sample of adult women from the USA, UK and Australia was recruited to complete an anonymous, online questionnaire. A total of 914 women, aged 18-87 years (*M* = 36.80, *SD* = 13.48) consented to take part in the study and completed at least one portion of the questionnaire. Table 1 presents a demographic summary of participants’ age, body mass index, education and ethnicity by country of residence. Demographic information was not available for women who did not report their country of residence as these questions were grouped together at the very end of the questionnaire.

**Table 1:** Participant demographics

	**USA**		**UK**		**Australia**		**Elsewhere**	
	**(*n*** **= 367)**		**(*n*** **= 224)**		**(*n*** **= 168)**		**(*n*** **= 22)**	
	*** M***	*** SD***	*** M***	*** SD***	*** M***	*** SD***	*** M***	*** SD***
*Age*	39.41	14.37	32.28	11.75	37.42	12.21	34.82	12.67
*Body Mass Index*	24.09	4.68	24.69	6.31	25.48	5.63	23.80	4.01
	n	%	n	%	n	%	n	%
*Education*^*a*^								
Some high school	2	0.5	0	0	5	2.60	0	0
High school	27	7.4	48	21.52	20	11.90	6	30.0
Diploma/ Associate’s Degree	20	5.4	23	10.31	35	21.21	0	0
Undergraduate	142	38.8	95	42.60	70	41.67	7	35.0
Postgraduate	175	47.9	57	25.57	38	22.62	7	35.0
*Ethnicity*^*b*^								
White	312	85.7	210	93.8	153	91.1	18	81.1
Other	52	14.3	14	6.2	15	8.9	4	18.1

a. Education indicates highest level of education completed. Frequencies (%) calculated with available data on a within country basis.

b. Because we had an international sample and the vast majority of participants identified as White, for brevity we collapsed all other ethnic groups into ‘Other’.

### Procedure

On authors’ request, community organizations and workplaces with large female populations (e.g., universities, YWCA) distributed a standardized email inviting women to complete a brief online questionnaire titled “Women’s Conversations and Opinions about Themselves”. Similarly worded invitations also were posted on internet forums (e.g., Mumsnet, BubHub) and social networking websites (e.g., Facebook) and emailed to authors’ contacts. All emails and posts requested that women forward the study invitation to their own social networks.

The recruitment email described the study purpose as exploring topics that women frequently discuss with other women and how this relates to their health and well-being. After providing consent, participants completed measures online in the order that they are described below. Upon completion, participants could enter a prize draw to win a £50/$80 Amazon gift voucher. Ethical approval to conduct the study was granted by the internal review boards at the authors’ respective institutions.

### Measures

#### Demographic information

Participants reported their age, height, weight, country of residence, highest level of education achieved and ethnicity. Body Mass Index (BMI) was calculated by dividing self-reported weight (kilograms) by height^2^ (meters). Although gathering participant’s height and weight data via self-report is not optimal, as this was a survey study we were unable to assess these constructs more objectively. Nonetheless, research has shown that self-report weights are reasonably accurate [30].

#### Fat talk

To assess the fat talk content, participants completed an imagined conversation script between themselves and a specific friend, in accordance with Salk and Engeln-Maddox [24]. The conversation began with a friend saying “*Ugh, I feel so fat*”. The participant then recorded her initial response to her friend in an open-ended response box, followed by a response from her friend and a final response from the participant.

To measure frequency of fat talk, participants received the following definition which included 9 fat talk examples (e.g., “she would be prettier if she lost weight,” “do I look fat in this?”): “*The term “fat talk” is used to describe any speech that implicitly or explicitly reinforces or endorses the thin-ideal standard of female beauty that is promoted in western culture. Fat talk can appear to be either critical (*e.g.*, “She’s too fat to wear that skirt”) or seemingly complimentary (*e.g.*, “I wish I was as thin as you”).*” Consistent with Salk and Engeln-Maddox’s methodology [24], participants next responded to three items on a 5-point Likert scale (1 = never or it’s extremely rare; 5 = it’s extremely common) to indicate how often they personally engage in fat talk, how often their female friends and family engage in fat talk, and how often they see or hear fat talk in the media.

We also administered a slightly modified version of the Fat Talk Scale [26]. This scale includes 9 scenarios in which a woman is talking to a female friend and fat talk arises (e.g., “*Anna and her friends are taking a workout class together. While changing into their workout clothes, one of Anna’s friends clutches her stomach and says she looks so fat. Her other friend says she hates her thighs. Anna responds with something she hates about her own body.*”). Participants indicated on a 5-point Likert scale (1 = never; 5 = always) the extent to which they would respond in the way Anna responded. We modified scenarios that appeared exclusively aimed at younger women (e.g., one that included walking to class) to be more age neutral. Reliability for the Fat Talk Scale in this study was good *(Cronbach’s α* = .92); previous research supports its test re-test reliability and validity [26].

#### Old Talk

To assess the old talk content, participants completed another imagined conversation script between themselves and a specific friend who begins by saying “*Ugh, look at these wrinkles. I can’t believe how old I look*”. The response format was consistent with the fat talk conversation task described above.

To measure frequency of old talk, participants received the following definition of old talk along with 9 old talk examples: *“The term “old talk” is used to describe any speech that implicitly or explicitly reinforces or endorses the young (and still thin) -ideal standard of female beauty that is promoted in western culture. Old talk can appear to be either critical (*e.g.*, “She’s looking really old.”) or seemingly complimentary (*e.g.*, “You so don’t look your age! Tell me your secret.”).”* Participants separately rated on a 5-point Likert scale (1 = never or it’s extremely rare; 5 = it’s extremely common) how often they personally engage in old talk, how often their female friends and family engage in old talk, and how often they see or hear old talk in the media.

Based upon the Fat Talk Scale [26], we created an Old Talk Scale for this study. This scale included 9 scenarios in which a woman named Anna is talking to female friends and old talk arises (see Appendix A for Old Talk Scale). Scenarios were based on conversations observed by the authors or pilates instructor mentioned above. Participants indicated on a 5-point Likert scale (1 = never; 5 = always) the extent to which they would respond as Anna did in each scenario. Higher scores indicate a greater tendency to engage in old talk. Reliability for the Old Talk Scale was good (*Cronbach’s α* = .88).

#### Thin-ideal Internalization

To assess thin-ideal internalization, the internalization-general (9 items; e.g., “I compare my body to the bodies of people who are on TV”; 1 = definitely disagree, 5 = definitely agree) and internalization-athlete (5 items; e.g., “I compare my body to that of people in "good shape”; 1 = definitely disagree, 5 = definitely agree) subscales of the Sociocultural Attitudes Towards Appearance Questionnaire-3 (SATAQ-3) [31] were administered. Higher scores indicate greater internalization of cultural beauty ideals. The SATAQ-3 is widely used and has good convergent validity [31]. Reliability for the subscales was good (*Cronbach’s α* = .93; .84).

#### Body areas satisfaction

The Body Areas Satisfaction subscale of the Multidimensional Body Self Relations Questionnaire (MBSRQ) [32] assessed women’s satisfaction with discrete body parts. Participants rated their satisfaction with 9 aspects of appearance (e.g., face, mid torso, weight) on a 5-point Likert scale (1 = very dissatisfied, 5 = very satisfied). Significant data supports the reliability and validity of the MBSRQ [32]. Higher scores indicate greater body satisfaction. Reliability was good in this sample (*Cronbach’s α* = .85)

#### Self-objectification

The Self-Objectification Questionnaire [33] assessed the extent to which women focus on appearance-based aspects of their bodies in comparison to competence-based aspects. Participants ranked body attributes in order of perceived importance to physical self-concept (1 = most important, 9 = least important). Scores were calculated by computing a difference score between the sum of the ranks for appearance-based items (e.g., physical attractiveness, weight) and those for competence-based items (e.g., fitness, health), with higher scores indicate greater levels of self-objectification. Previous research has demonstrated that this scale has acceptable construct validity [33].

#### Ageing appearance anxiety

The Physical Appearance subscale of the Anxiety about Aging Scale [34] assessed concern about the effects of ageing on appearance (5 items; e.g., “When I look in the mirror, it bothers me to see how my looks have changed with age; 1 = strongly disagree, 5 = strongly agree). In our review of the aging literature, we found this to be the most widely used scale. Lasher and Faulkender [34] found good internal consistency for this measure, which has high face validity. Higher scores indicate greater ageing anxiety and the scale had good reliability in this study (*Cronbach’s α* = .77).

#### Drive for thinness

The Drive for Thinness subscale from the well validated Eating Disorder Inventory-2 [35,36] assessed participants’ preoccupation with weight and dieting (7 items; e.g., “I think about dieting”; 1 = never, 6 = always). Higher scores indicate greater drive for thinness. The scale had good reliability in this sample (*Cronbach’s α* = .83).

#### Eating disorder pathology

Diagnostic items from the Eating Disorders Examination-Questionnaire [37] assessed ED pathology (12 items; e.g., “Over the past 28 days, how many times have you made yourself sick (vomit) as a means of controlling your shape or weight?”). Higher scores indicate greater eating pathology. Research supports the two week test-retest reliability, internal consistency, and temporal stability of the EDE-Q [38,39]. The scale had good reliability (*Cronbach’s α* = .79).

## Results

Because our sample was substantially more geographically diverse than other studies in this area, we examined if there were any differences between the main countries in our study prior to treating the sample as a unified group. For this analysis, we removed participants who resided in a country other than the USA, UK or Australia (*n* = 22), as well as participants who did not report country of residence (*n* = 133). Preliminary analyses indicated that countries differed with respect to age and BMI, thus we co-varied these variables in the between-country analyses. Results indicated that participants from all countries were equally likely to engage in fat talk, *F* (2, 756) = 2.30, *p* = .831, *η*_*p*_^*2*^ = .006. We similarly found no significant differences in reported old talk engagement and participant’s country of residence, *F*(2, 730) = 2.50, *p* = .084, *η*_*p*_^*2*^ = .007.

### Fat talk content

Responses to the fat talk conversation were coded and analyzed thematically by the third and fourth author in accordance with the procedure outlined by Salk and Engeln-Maddox [24]. To determine if the seven themes observed by Salk and Engeln-Maddox [24]. (*denial, empathy, probing, evidence, causes, action together, I’m fat you’re not*) captured the content of our data, we attempted to code conversations from the first 70 participants into these themes. Overall, the themes fit the data well (i.e., most responses were easily coded into the original seven themes), although three new themes were identified; *healthy ideal*, *disengagement* and *discount*. Table 2 provides a summary of the analysis of fat talk conversations.

**Table 2:** Fat talk content thematic analysis summary

**Theme**	**Definition**	**Example responses**	**Initial responses %**	**Entire conversation %**	***Kappa***
Denial	Participant denies that friend is fat	*“You’re not fat at all.”*	63.9	64.75	.85
Empathy	Participant indicates that she or others can relate to feeling fat and being upset	*“Ah I know how you feel.” “I can totally understand the feeling.”*	12.65	23.15	.78
Probing	Participant questions friend as to why she feels fat	*“What makes you feel like this?” “Why what’s wrong?”*	13.10	15.75	.84
Evidence	Friend provides evidence to support the statement that she is actually fat	*“Yes I am. Look at this (then grabs her love handles).”*	-	50.5	.70
Causes	Participant discusses or inquires about the causes contributing to friend’s belief	*“You need to stop watching these dumb rom coms.” “Have you not been exercising lately? Or is it PMS?”*	-	11.0	.82
Action	Participant suggests or prompts plan of action	*“Well have you tried eating better?”*	-	26.7	.84
I’m fat, you’re not	Participant disagrees by commenting on her own fatness	*“I don’t think you are fat, but I am.”*	-	6.75	.75
Healthy Ideal	Participant actively promotes health instead of appearance	*“The important thing is that you feel healthy and happy.”*	-	4.5	.77
Discount	Participant or friend rejects the other’s denial that they are not fat	*“Whatever, you’re only saying that because you have to.”*	-	28.7	.89
Disengage	Participant actively dismisses and attempts to stop the fat talk	*“Don’t say that!”*	-	13.0	.90

Note: Cohen’s kappa coefficients reported here were calculated for the analysis in which the coders analyzed the entire conversation. Kappa for initial response coding was .81.

The coders next analyzed all data by first coding participants’ initial responses into one of three dominant themes (*denial*, *probing*, *empathy).* The most common initial response to a friend engaging in fat talk was *denial* (63.9%), followed by *probing* (13.1%) and *empathy* (12.65%). A minority of initial responses (10.35%) did not fit into these themes. Inter-rater reliability (IRR) for the coding of initial responses was good (*Cohen’s kappa* = .81).

Secondly, the entire conversation for each participant was coded into the full set of 10 themes, with each conversation being coded into as many relevant themes as needed to adequately describe the data. The most common response types across the entire conversation were *denial* (64.75%), *evidence* (50.5%), *discount* (28.7%), *action (*26.7%) and *empathy* (23.15%). IRR was acceptable for all themes (*Cohen’s kappa* ≥ .70).

### Frequency of fat talk

As hypothesized, an overwhelming majority (81%) of the total sample reported engaging in at least occasional fat talk. Among the 881 who responded to this item, 33% reported frequently engaging in fat talk (score 4 or 5; see Table 3). The mean rating of fat talk was 2.86 (*SD* = 1.23).

**Table 3:** Frequencies and means for fat talk and old talk

	**None/Rare**	**Occasionally**	**Sometimes**	**Quite**	**Extremely**	***M *** **( *SD *)**
				**often**	**common**	
	**%**	**%**	**%**	**%**	**%**	
Self Fat Talk						
Total Sample	16	26	25	22	11	2.86 (1.24)
18-29 Years	15	23	24	23	15	2.99 (1.30)^a^
30-45 Years	15	28	24	24	10	2.86 (1.21)^ab^
46-60 Years	18	28	21	21	11	2.79 (1.29)^ab^
61+ Years	16	35	35	12	2	2.49 (0.98)^b^
Underweight	19	24	21	14	21	2.98 (1.44)^ab^
Normal Weight	16	30	24	21	9	2.77 (1.21)^a^
Overweight	9	25	27	24	15	3.12 (1.21)^b^
Obese	21	18	24	25	11	2.88 (1.25)^ab^
F&F Fat Talk	5	20	27	37	12	3.33 (1.06)
Media Fat Talk	1	5	12	38	45	4.21 (0.89)
Old Talk						
Total Sample	34	30	22	12	3	2.21 (1.12)
18-29 Years	52	29	15	3	1	1.17 (0.89)^a^
30-45 Years	27	29	24	24	4	2.40 (1.16)^b^
46-60 Years	14	31	30	17	7	2.71 (1.11)^c^
61+ Years	9	33	33	26	0	2.72 (0.94)^c^
F&F Old Talk	16	35	28	17	4	2.57 (1.07)
Media Old Talk	3	14	24	39	20	3.59 (1.06)

Note: Subgroupings with different superscripts differ significantly from one another at *p* < .05. Subgroupings with the same superscripts did not differ significantly from one another. *F&F Fat Talk*, Friends and Family Fat Talk. *F&F Old Talk*, Friends and Family Old Talk.

Because our sample included a much more diverse range of ages and weights than most previous studies of fat talk, we examined if these factors were associated with differences in fat talk. We divided participants into four main age groups (18-29 years, *n* = 292; 30-45 years, *n* = 293; 46-60 years, *n* = 141; and 61 and older, *n* = 55) and excluded those who did not report age (*n* = 133). Groups were created with the following concerns in mind: not wanting to create too many groups, having ages that roughly held together in terms of life stages, and not creating groups with very small samples. We also consulted a life span developmental psychologist for advice in creating the groups as well as the lifespan literature [40]. Preliminary analyses indicated that BMI differed for the different age groups. Thus we co-varied BMI.

In contrast to our hypothesis, we found a significant difference in frequency of engaging in fat talk based on age, *F* (3, 753) = 3.98, *p* = .008, *η*_*p*_^*2*^ = .016. Mean ratings indicated that engagement in fat talk slowly decreased as participant age increased (see Table 3), although rates of at least occasional fat talk remained stable across age group (82-86%). As can be seen in Table 3 fat talk did not noticeably change until women reached the oldest age bracket (> 61 years), and post-hoc analyses indicated that only the youngest and oldest age groups significantly differed. Thus our hypothesis that fat talk would remain largely unchanged across age groups was mostly, but not fully, supported.

To analyze differences by weight, we used traditional BMI cut-off points to split our sample into underweight (BMI <18.5, *n* = 42), normal weight (BMI = 18.5-24.9, *n* = 452), overweight (BMI = 25-29.9, *n* = 158) and obese categories (30+, *n* = 115). See Table 3 for frequencies and means. Preliminary analyses indicated that BMI groups differed with respect to age, which we co-varied. Results indicated that BMI groups differed significantly in self-fat talk frequency, *F* (3, 753) = 4.02, *p* = .007, *η*_*p*_^*2*^ = .016. Mean ratings indicated that the overweight group engaged in the most fat talk whereas the normal weight group engaged in the least. Post-hoc analyses indicated that only these two groups differed significantly, and an overwhelming majority of all groups reported at least occasional fat talk (79% - 91%). Thus our hypothesis that fat talk would occur equally across women of all weights was largely, but not fully, supported.

Given the missing age data for 133 participants, the fact that most sub-groups did not differ from one another in age and BMI analyses, and our desire to avoid over-analyzing our data, we examined reported frequency that female friends and family engage in fat talk (F&F FT) and media fat talk for the total sample only. For F&F FT, 96% of participants responding to this question (*n* = 878) reported at least occasional fat talk. As hypothesized, we found a third person effect, with mean F&F FT frequency being greater compared to self, *t* (877) = 11.81, *p* < .001. For media fat talk, 99% of participants reported exposure to at least occasional media fat talk. Consistent with our third person effect hypothesis, mean fat talk significantly rose from self to media, *t* (878) = 28.36, *p* < .001.

### Old talk content

Responses to old talk conversation were coded and analyzed thematically in accordance with the procedure described above for fat talk. To determine if the ten themes observed in the fat talk conversations (*denial, empathy, probing, evidence, causes, action, I’m old you’re not, healthy ideal, discount* and *disengage*) captured the content of the old talk conversations, we attempted to code the old talk conversations from the first 70 participants into these themes. Overall, the themes fit the data well (i.e., most responses were easily coded into the 10 fat talk themes); *probing* and *healthy ideal*, however, occurred in a minority of cases (< 2.15%) and were therefore removed. Additionally, one new theme was identified; *ageing is natural and positive.* Table 4 provides a summary of the analysis of old talk conversations.

**Table 4:** Old talk content thematic analysis summary

**Theme**	**Definition**	**Example responses**	**Initial responses %**	**Entire conversation %**	***Kappa***
Denial	Participant denies that friend is old	*“You don’t look old at all!”*	60.65	51.5	.84
Empathy	Participant indicates that she and others can relate to feeling old and concerned about ageing appearance	*“I know, I feel the same, it just catches up with you when you aren’t looking and it’s only going to get worse.”*	13.45	14.75	.75
Ageing is natural and positive	Participant implies that ageing is natural, positive and/or inevitable	*“Aging is natural and beautiful.... I wish I had wrinkles, they make you look wise.*	18.15	28.7	.75
Evidence	Friend provides evidence to support the statement that she is actually old	*“I disagree, LOOK at them.”*	-	35.35	.74
Causes	Participant discusses or inquires about the causes contributing to friend’s belief that she is old and showing visible signs of ageing	*“It’s from smiling and laughing too much”*	-	4.35	.92
Action	Participant suggests or prompts plan of action to address feelings	*“If you’re so worried, get some decent anti-wrinkle cream, nothing to lose!”*	-	15.2	.80
I’m old, you’re not	Participant disagrees by commenting on her own signs of ageing	*“That is so not bad. Look at my wrinkles”*	-	8.9	.84
Discount	Participant or friend rejects the other’s denial that they are not old	*“Yes I do, look at these!”*	-	23.75	.71
Disengage	Participant actively dismisses and tries to stop the old talk	*“Girl, don’t even go there. You are gorgeous.”*	-	5.85	.90

Note: Cohen’s kappa coefficients reported here were calculated for the analysis in which the coders analyzed the entire conversation. Kappa for initial response coding was .83.

The coders then analyzed the full dataset by first coding participants’ initial responses into one of three dominant themes (*denial*, *empathy, ageing is natural and positive).* The most common initial response to a friend engaging in old talk was *denial* (60.65%), followed by *ageing is natural and positive* (18.15%) and *empathy* (13.45%). A minority of initial responses (7.75%) did not fit into these themes. IRR for initial response coding was good (*kappa* = .83).

The entire conversation for each participant was then coded into the full set of nine themes. The most common response types across the entire conversation were *denial* (51.5%), *evidence* (35.35%), *ageing is natural and positive* (28.7%), *discount* (23.75%) and *action (*15.2%). IRR was acceptable for all themes coded across entire conversations (*Cohen’s kappa* ≥ .71).

### Frequency of old talk

We repeated the major fat talk analyses for old talk frequency, with the exception of the BMI analyses because that was not part of our original set of hypotheses or research design. Of the 830 participants who responded to the self-old talk question, as hypothesized, the majority of women (66%) reported engaging in old talk (see Table 3). Although this is lower than that for fat talk (also consistent with our hypotheses), it still represents the majority of participants. In the total sample, the rate of frequent old talk (score 4 or 5) was lower (15%) than the rate of frequent FT (33%). The mean rating for self-old talk was 2.21 (*SD* = 1.12).

As hypothesized, the ANCOVA for age, *F* (3, 753) = 46.33, *p* < .001, *η*_*p*_^*2*^ = .015, was highly significant. Frequency of old talk steadily increased as participant age increased. Additionally, the oldest two age groups reported engaging in old talk at a similar or greater frequency than fat talk (see Table 3). Post-hoc tests indicated that the youngest group differed significantly from each of the three older age groups (in ascending order: *t* (583) = −8.04, *p* < .001; *t* (431) = −10.05, *p* < .001; *t* (345) = −7.81, *p* < .001), and that the second youngest age group differed from the two oldest age groups (in ascending order, *t* (432) = −2.62, *p* = .009; *t* (346) = −2.03, *p* = .04). In summary, our hypothesis that frequency of old talk increases with age was supported. Surprisingly, however, almost 50% of the youngest age group also reported engaging in at least occasional old talk.

With regards to friends and family old talk (F&F OT), an overwhelming majority (84%) of participants reported at least occasional F&F OT, and we found a significant third person effect, *t* (826) = −10.88, *p* < .001. As with fat talk, we see a pronounced rise in frequency in response to the media old talk question. Ninety-seven percent of participants reported exposure to at least occasional media old talk, and 59% frequent. Media old talk differed significantly as compared to self-old talk, *t* (829) = −29.56, *p* < .001.

### Relationships between fat talk, old talk, body image and ED pathology

The mean fat talk and old talk scale scores were 21.61 (*SD* = 8.13) and 15.8 (*SD* = 6.28) respectively. As hypothesized, scores on the fat talk and old talk scales were significantly, but *not* perfectly, correlated, *r* = .54, *n* = 776, *p* < .001 (two tailed: see Table 5). This suggests that fat talk and old talk are related, but distinct phenomena. Also as hypothesized, the fat talk scale was significantly correlated with thin-ideal internalization (both general and athlete), body areas satisfaction (negative correlation), self objectification, physical appearance aging anxiety, drive for thinness and ED pathology (*r’*s range from .23-.67; all *p* < .001; see Table 5). The same was found for the old talk scale (*r’*s range from .23-.47; all *p* < .001; see Table 5). Consistent with our hypothesis, correlations for the fat talk scale were larger than those found for the old talk scale, with the exception of the appearance ageing anxiety.

**Table 5:** Correlations between fat talk & old talk scales and measures of body image and eating disorder pathology

	**Total sample**		**Ages 46+**	
	**Fat talk scale**	**Old talk scale**	**Fat talk scale**	**Old talk scale**
Old Talk Scale	.535*		.719*	
SATAQ-3 General Intern	.497*	.331*	.452*	.459*
SATAQ-3 Athlete Intern	.316*	.271*	.312*	.346*
Body Areas Satisfaction^a^	-.528*	-.385*	-.522*	-.402*
Self Objectification	.441*	.232*	.430*	.340*
Appearance Ageing Anxiety	.230*	.474*	.312*	.461*
EDI Drive for Thinness	.668*	.387*	.629*	.513*
EDE-Q ED Pathology	.540*	.385*	.612*	.603*

a Body Areas Satisfaction: Higher scores are better. * *p* < .001.

Based on the finding that old talk frequency was most common in the two oldest age groups, we also examined correlations in this subsample (see Table 5). As with the total sample, all correlations were highly significant (*p* < .001). Interestingly, the fat talk and old talk scales became more correlated in this subgroup. Further, in contrast to the total sample, old talk correlations were mostly equivalent to, or greater than, those found for fat talk for thin-ideal internalization (general and athlete) and ED pathology. Finally, with the exception of appearance ageing anxiety, all correlations increased for the old talk scale in this subsample relative to the general sample.

## Discussion

Research indicates that body dissatisfaction is associated with a range of physical and mental health problems. As such, identifying factors that are associated with and may play a role in causing, sustaining, or deepening body dissatisfaction is an important public health issue. Even more critical is the identification of factors that are modifiable and therefore potentially important targets for body image interventions. Previous research implicates fat talk as a factor that not only is associated with body dissatisfaction and ED pathology, but also increases body dissatisfaction when experimentally manipulated. This study contributes to the existing literature by exploring fat talk among women from three countries across the lifespan. Our findings also suggest that researchers need to broaden their study of body image talk to include old talk, particularly when studying midlife and older adult women.

In contrast to existing literature that has typically focused on fat talk as a behavior of adolescents and young women, we found that women of all ages reported engaging in at least some fat talk, with rates of occasional fat talk remaining at a constant 82-85% across our four age groups. Although frequency of fat talk did gradually decrease in the older age groups, the reduction was non-significant except when the youngest and oldest groups were compared. These results suggest that fat talk research needs to include, at minimum, women through the full midlife stage. Moreover, because no body image study has yet followed midlife and older women over a significant period of time, it remains unclear to what degree current findings represent cohort effects. More specifically, it may be that lower rates of fat talk in the very oldest age group represent a generation difference and that women who are currently in their 20s will sustain their current levels of fat talk as they age.

Results for old talk across the ages showed a different pattern than those for fat talk. First, as hypothesized, overall rates of old talk were lower than that for fat talk, with 66% of women reporting at least occasional old talk. Second, whereas rates of fat talk slowly decreased as women aged, rates of old talk increased over the lifespan. The different trajectories of fat talk and old talk across the lifespan provide support for the notion that fat talk and old talk are distinct constructs. They also suggest that when women are young, the most salient feature of the thin-young-ideal is thinness. In contrast, when women are in mid-life both thinness and youth appear to be of concern. Finally, when women enter older adulthood youth appears to become more salient than thinness. Having said this, fat talk was still common among the eldest sub-group, and almost 50% of the youngest age group reported periodic old talk. This suggests that in all ages we should examine effects of the thin-young-ideal, and not just focus on the thin aspect of ideal female beauty.

In contrast to our hypothesis that there would be no differences in fat talk based on body weight, we found a significant difference based on BMI. Although women across all weight categories reported high levels of at least occasional FT (79-91%), frequency of fat talk was highest in our overweight group, which differed significantly from the normal weight group. This contrasts to research with younger women and adolescent girls. Nichter [22] described fat talk as something that almost exclusively occurs among underweight and normal weight adolescent girls. Our findings are, however, consistent with the only other study to examine adult women. Martz et al. [29] found that obese participants reported the greatest pressure to engage in fat talk.

As hypothesized, we found a third person effect [41] for the perceived extent to which both friends and family and the media engage in both fat talk and old talk. This means that women are likely to report that other people engage in significantly more fat talk and old talk then they themselves do. Rates for media fat talk and media old talk were both extremely high. In contrast to extensive research examining effects of idealized media images on body image and health, there is little research that has examined media fat talk and none for media old talk. Given findings of Stice and colleagues [27], in which exposure to a low dose of fat talk by a confederate produced significant worsening of body satisfaction, we suggest that research is needed to determine effects, if any, of media fat talk and media old talk.

As expected, fat talk and old talk scales were significantly correlated with each other, albeit not perfectly. Again, this suggests that the scales tapped into related but different constructs. Both scales also were significantly correlated with all body image and ED pathology measures included in this study. Women who reported greater levels of fat talk and old talk were also more likely to report higher levels of thin-ideal internalization, self-objectification, appearance ageing anxiety, drive for thinness and ED pathology as well as decreased body satisfaction. As hypothesized, fat talk was more strongly related to all body image and ED measures, with the exception of the ageing appearance anxiety, which was more highly correlated with the old talk scale. When we examined the same correlations among women 46 years and older, who reported engaging in the most old talk, we found that almost every correlation for old talk increased. This suggests that old talk is more strongly linked to body image and ED disturbance when it is examined in the population for which it is most salient.

This study has several limitations. First, we used convenience, snowball sampling versus probability samplings. Second, a majority of the women were under 45 years of age. Third, our reliance on internet sampling means the study could have selection bias particularly with respect to socio-economic status (SES) as internet access may be a proxy for SES. Fourth, we relied on self-report. Although this methodology is common in fat talk studies, future research should include other methodologies.

## Conclusion

This study provides a significant contribution regarding body image talk among women, and suggests that, due to its prevalence and association with body image and ED pathology, old talk should be addressed in future body image research, particularly among women in mid-older life. Additionally, results suggest that body image programs targeting mid-life and older women may benefit from addressing a) the thin *and* young aspects of idealized female beauty and b) the role of old talk and fat talk in perpetuating unhelpful beauty ideals and appearance-related distress. Finally, results suggest that calls for greater body size diversity in media imagery to promote positive body image (e.g., Australian Government [42]) should also be accompanied by calls to reduce discourse that stigmatizes and reinforces unhelpful beauty ideals in relation to age.

## Appendix A

### Old talk scale

Below are some scenarios in which women express and respond to aging concerns. Please read each scenario and then answer the corresponding question* by circling Never (=1), seldom , sometimes, often, or always (=5).

(*The corresponding question for each item is “Please indicate the extent to which you would respond the way Anna did in this situation.”).

1. Anna is having a bad day. She does not feel herself and is somewhat down. While walking to a meeting, a female co-worker says that she looks nice today. Anna groans and says “You obviously aren’t looking at my face - I think I developed new wrinkles overnight. I look so old and tired.”

2. Anna and her friends are taking a workout class together. While changing into their workout clothes, one of Anna’s friends looks in the mirror and says “I really need to start Botox.” Her other friend says she is thinking about getting her teeth whitened and dermabrasion to even out her skin tone. Anna responds with something she hates about her own body.

3. Anna is eating lunch with her friends and decides to have dessert. As she finishes her dessert, she makes a comment like “I can’t believe I did that. My belly is so huge compared to when I was younger. I hate it but I can’t seem to fix it.”

4. Anna and her friend are walking to their car after watching a movie. Anna comments that the lead actress looks amazing for her age and says “I wish I could look even half that good these days. I wonder what she does to look that good. I hate getting older.”

5. Anna is shopping with her friend Emily. Anna is at the cosmetics counter and asks Emily “what do you use on your skin. I need something that will make me look younger. I keep trying different things but nothing works.” Emily suggests checking out a recent fashion magazine review of anti-aging products.

6. Anna is hanging out with a friend when she looks in the mirror and says “I don’t think I can wear a swimsuit this year. I have developed a middle aged body and I just can’t seem to accept it.”

7. Anna is talking with a friend about dating. Anna says “I might as well give up. No one wants to go out with someone as old as me.” Anna’s friend responds she is thinking of getting plastic surgery because “a facelift will hurt less than seeing John start looking at younger girls.”

8. Anna is taking a workout class with a friend when a substitute teacher enters the room. Anna’s friend states - “That can’t be our teacher - she’s too old to be teaching a class like this.”

9. Anna’s friend Emily really feels as though she looks older than she likes. Emily turns to Anna and says “This is a secret, but I am really thinking about getting a face and neck lift. I think it’s the only way I am going to feel better about myself.” Anna says “I know what you mean. I don’t feel old. I just wish I looked as young as I feel.”

How old did you think Anna was as you completed this scale?

## Competing interests

The authors have no competing interests to report.

## Author contributions

CBB created idea for study, collaborated in the study design, analyzed quantitative data, wrote a majority of the first draft, and edited the manuscript. PD collaborated in the study design, created and managed the online survey, cleaned the data, supervised GJ and CW in the coding and analysis of the qualitative data, contributed to the writing of the first draft, and edited the manuscript. GJ collaborated in the study design, coded and analyzed the qualitative data, contributed to the writing of the first draft and edited the manuscript. CW coded and analyzed the qualitative data, contributed to the writing of the first draft and edited the manuscript. All authors read and approved the final manuscript.
